# Towards IoT-Aided Human–Robot Interaction Using NEP and ROS: A Platform-Independent, Accessible and Distributed Approach

**DOI:** 10.3390/s20051500

**Published:** 2020-03-09

**Authors:** Enrique Coronado, Gentiane Venture

**Affiliations:** Department of Mechanical Systems Engineering, Tokyo University of Agriculture and Technology, Tokyo 184-0012, Japan; venture@cc.tuat.ac.jp

**Keywords:** internet of robot things, artificial intelligence and robotics, human–computer interaction, human–robot interaction

## Abstract

This article presents the novel Python, C# and JavaScript libraries of Node Primitives (NEP), a high-level, open, distributed, and component-based framework designed to enable easy development of cross-platform software architectures. NEP is built on top of low-level, high-performance and robust sockets libraries (ZeroMQ and Nanomsg) and robot middlewares (ROS 1 and ROS 2). This enables platform-independent development of Human–Robot Interaction (HRI) software architectures. We show minimal code examples for enabling *Publish/Subscribe* communication between Internet of Things (IoT) and Robotics modules. Two user cases performed outside laboratories are briefly described in order to prove the technological feasibility of NEP for developing real-world applications. The first user case briefly shows the potential of using NEP for enabling the creation of End-User Development (EUD) interfaces for IoT-aided Human–Robot Interaction. The second user case briefly describes a software architecture integrating state-of-art sensory devices, deep learning perceptual modules, and a ROS -based humanoid robot to enable IoT-aided HRI in a public space. Finally, a comparative study showed better latency results of NEP over a popular state-of-art tool (ROS using rosbridge) for connecting different nodes executed in local-host and local area network (LAN).

## 1. Introduction

**Figure 1:**
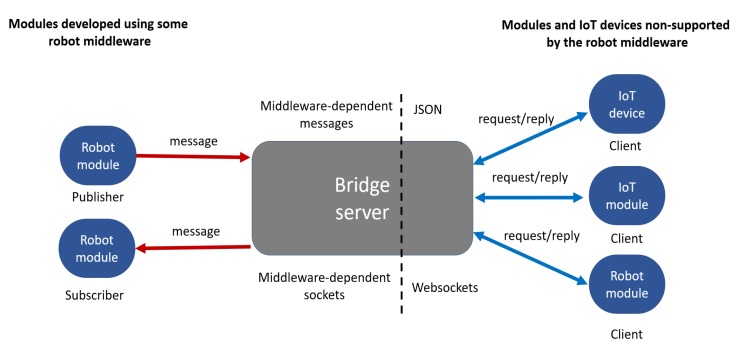
Example of robot and Internet of Things (IoT) components connected with non-robot-middleware-capable devices and modules via a bridge server.

The rapid development of sensors, devices, and algorithms in Robotics is nowadays engaging new users outside its classical industrial and academic technological communities [1,2]. To be adopted by a broader community and accessible to more people, robots must be able to communicate with user’s devices (e.g., computers, tablets, smartphones, and smartwatches) for monitoring, programming and control tasks. In fact, enabling this connection using modern communication technologies is one of the main objectives of the Internet of Robot Things (IoRT) [3]. In this context, the availability of robot software compatible with the resources (i.e., devices and software) of different types of users becomes relevant for supporting their research and work activities. However, many academic-oriented software and distributed frameworks for robot programming and application development are designed to exclusively or better work in desktop computers with some Linux-based desktop Operating Systems (OS), such as Ubuntu [4]. This limits accessibility for many experts and novice users preferring or constrained to use other OS. Moreover, many cross-disciplinary projects can require high-performance, easy and robust communication between software modules written in different programming languages. This is often done to increase the robot’s capabilities and functions using novel sensors and algorithms. However, most robotic frameworks tend to officially support a few programming languages, generally being C++ the main available option according [4]. Relevant state-of-art robot frameworks are YARP [5] (developed in C++), ROS [6] (officially supporting C++, Python 2 and Lua) and ROS 2.0 [7] (only supporting C++ and Python 3). To communicate with non-supported devices and programming languages, some robotics middleware/frameworks integrates bridge servers. As explained in Figure 1, this approach transforms middleware-dependent messages coming from middleware-dependent sockets to messages serialized in some format which non-supported devices or software modules can read and write. A popular serialization and data streaming format is the Javascript Object Notation (JSON). These messages are generally transmitted using the *Request/Process/Reply* communication pattern (also denoted *Client/Server*) via POSIX sockets or *Websockets*. The module that performs this transformation between protocols is often called a bridge server. Relevant state-of-art packages is the rosbridge suite [8] and *yarp.js* [9]. As described in the official ROS documentation [10], rosbridge is composed of two parts: the protocol and the implementation. On the one hand, the rosbridge serialization protocol is a specification for sending JSON messages to ROS. On the other hand, the rosbridge implementation, also denoted rosbridge suite, is a collection of packages that implement the rosbridge protocol over *Websockets*. These packages include the rosbridge library, *rosapi* and rosbridge server. Any future reference to “rosbridge” in this article is made over the rosbridge implementation and not about the rosbridge protocol. In [9], *yarp.js* is defined as “a JavaScript framework enabling robotics networks to interface and interact with external devices by exploiting modern Web communication protocols”. Similar to the rosbridge suite, *yarp.js* also requires the use of a bridge server implementation [9].

However, the use of the *Client/Server* pattern tends to create less reliable software architectures due to communication issues (e.g., loss of connection and deadlocks) as well as lower performance for data streaming when comparing with the *Publish/Subscribe* pattern [11,12]. These issues can be trivial for short-term and structured experiments requiring the streaming of a low volume of data at relatively low rate. However, they become relevant when building complex HRI applications requiring: (i) the streaming of high volume of data and sensory information at high rates, and (ii) to be performed in long-term and unstructured Human–Robot Interaction (HRI) studies outside laboratories. Moreover, solutions such as rosbridge in ROS 1.0 and *yarp.js* force non-Linux users to acquire and configure an additional computer with Ubuntu to enable the execution of the bridge server from which all messages need to go through [8,9].

This paper explains how the Node Primitives (NEP) robot programming framework can be used for enabling user-friendly development of cross-platform and distributed IoT-aided Human–Robot Interaction software architectures and applications. NEP has been tested in many versions of Windows, Linux and OSX, including those currently not fully compatible or still not well supported by the ROS frameworks, such as Windows 7, 8.1 (not supported by ROS 2.0) and 10 (not fully supported by ROS 1.0). NEP has also been designed to provide lightweight, simple, high-performance, usable and easy-to-install, inter-process communication and monitoring tools. In addition, NEP provides a high-level abstraction in Python, which enables the easy re-use of code between different middlewares. Current back-end options for Python include ROS 1.0 and ROS 2.0, ZeroMQ [13], POSIX sockets and Nanomsg [14]. In other programming languages, NEP runs on top of ZeroMQ. The proposed libraries in Python, JavaScript, and C#, which are the main contributions of this article, enables the easy connection between software modules coming from different Information Technologies (IT) disciplines. The technological feasibility of the proposed software is proved in two real-world applications using both a non-ROS-based robot (Pepper) and a ROS-based robot (Open Next Stage).

This article is organized as follows. Section 2 discusses related works and highlights some of the main differences between NEP and popular current state-of-art solutions. Section 3 presents the main contributions of this article and shows some minimal code examples. Section 4 briefly presents real-world IoT-aided HRI applications developed with NEP. Finally, Section 4 presents a comparative study of NEP using ZeroMQ against solutions based only in rosbridge and ROS 1. Conclusions and future work follow.

## 2. Background

### 2.1. Related Work

Even when there is a clear dominance of ROS frameworks as the main communication tool for the integration of academic software in robotic laboratories, it is not totally free of disadvantages. As described in [15], ROS and most robot middleware still present many shortcomings regarding usability, portability, accessibility, compatibility and platform dependencies. The authors of [16] also describe the difficulty in learning as one of the main reasons why users do not prefer or use ROS frameworks for their robotic projects. In fact, many industrial and short-term academic projects can require the use of easy-to-use, install and learn tools to enable the integration of the robot modules. Therefore, the development of alternative robot middlewares and distributed robot programming software is still a trending topic in the robotics community. Examples of some recent ROS alternatives are described in [4,11,17,18,19]. As happens with almost all software, a solution can difficulty be better suited for all types of applications or desired by all types of users. Therefore, rather than trying to re-invent the wheel or directly compete with ROS, these alternatives are often designed to better support some specific users and applications where ROS-based frameworks do not fully satisfy their requirements. Probably, the most relevant ROS alternative is the YARP ter [5] framework, which has been designed to be the main distribution platform for the iCub robot [20]. Unlike ROS 1.0, which middleware capabilities were developed from scratch, YARP was developed on top of the Adaptive Communication Environment (ACE) library [21,22]. Recently, ROS 2.0 has been launched for mostly satisfying industrial requirements. Rather than be developed from scratch, ROS 2.0 is built on top of Data Distribution Services (DDS). Unfortunately, ROS 2.0 is still in its initial development *phases/releases*; therefore providing less usability and software tools when compared with ROS 1.0. Moreover, ROS 2.0 requires the installation of many-third party libraries for its execution in Windows.

### 2.2. Motivations

Most robotics experiments and projects up to date are performed in advanced robotics laboratories populated by many IT experts and high-end computers [23]. A typical motivation of these academic projects is to overcome the technological performance reached by competitors. This approach denoted as *machine-centered* design is generally the main focus of academic and technological communities in the initial years of research and development of novel technological devices [24,25]. In many projects performing *machine-centered* research, the use and installation of complex and academic-oriented software frameworks and Linux-based OS are often considered trivial and preferred tasks. This situation often differs when developers require to work together with non-IT experts that are interested to use robots for creating novel experiences (e.g., designers, social researchers, artists, sellers) [2]. In this article, these novice or general users of computers and robots are referred to as *end users* [26]. The inclusion of *end users* in research activities must also take into account the resources, capabilities, needs, and desires of humans by providing usable, portable, accessible, secure and aesthetic products [27,28]. This approach often denoted as *human-centered* design is an essential step to reach maturity and economical success when developing technological products for the general user [24,29,30]. A recent *human-centered* trend in HRI research in to bring robotic research activities, often done in laboratories, to *“in the wild”* (i.e., natural and every-day settings) and unstructured scenarios [1,23]. Research activities of HRI “in the wild” require to bring robots to new, open, noisy, crowded and natural scenarios outside laboratories, industrial and advanced professional settings (e.g., application for search and rescue and remote operation) [23,31]. In many cases, these applications must be conducted by novice *end users*, which can also require to install, set-up and update robot applications by themselves using their own computers. Therefore, this article presents a novel, cross-platform and lightweight robotic framework aimed to support *human-centered* IoT-aided HRI applications and “in the wild” research tasks. For this, the presented framework (denoted as NEP) provides accessible, portable and easy-to-use inter-process communication tools for robotics.

### 2.3. What Is NEP?

*NEP is a high-level framework*. Similarly to Keras [32], which is capable of running on top of TensorFlow [33] and Theano [34] for easy and fast prototyping of Deep Learning tasks, NEP is a high-level communication framework capable of running on top of low-level message libraries and robotic middlewares to provide simple and user-friendly development of accessible, re-usable and cross-platform robotics applications. This is done by enabling the communication between sensory, perceptual and cognitive robotics processes denoted as nodes (following the same terminology that ROS). These nodes, which are often written in different programming languages, can be executed on the same computer or in different computers and smart-devices connected at the *same cluster or IoT local network*. Unlike ROS, which is often used to handle low-level data generally defined in common structures or messages (e.g., force, torque, velocity, pose), NEP has been originally designed to deal with high-level data where there is not a unique or common way of representation (e.g., emotions, speech, gestures, behaviors, and objects). Therefore, messages in NEP are mostly defined using the JSON, which is a human-readable standard for the interchange of data. JSON is not only the preferred data interchange format for web services and applications, but also is highly used in many IoT applications [35,36,37]. However, NEP can also be used to send low-level and sensory data representations such as images.

*NEP is a Robotics platform support.* According to the definition classification done in [38] NEP is a IoRT robotics platform. Unlike ROS and YARP that provide a large variety of tools and algorithms for low-level development in robotics (e.g., simulators, inverse kinematic and planners), NEP focuses only on communication and the monitoring of data. This makes NEP easy-to-install using wizards (for *end-user* software distribution) or popular package manager (for developers), such as *pip*, *nuger* and *npm*. In the default configuration of NEP, links between modules can be managed by a ROS-like Master node (more details in [6,16]). This approach enables the creation of large, flexible and scalable applications. However, sockets connections (IP address and ports) in NEP can also be directly defined by the user, similar to YARP, for simpler and robust communication between modules. When reusing NEP code in ROS 1.0 and ROS 2.0 (i.e., selecting them as back-end options), links between modules are managed by the ROS Master and the installed Data Distribution Service (DDS) respectively.

*NEP uses mainly ZeroMQ*. The default back-end option of NEP is ZeroMQ because it provides a lightweight, portable and high-performance library for inter-process communication that can be easily installed in almost all programming languages and OS. Furthermore, the creation of ROS-like peer-to-peer (P2P) communication using topics between components using ZeroMQ and Nanomsg can be a difficult and time-consuming task requiring the manual configuration of sockets and the definition of the serialization approach (i.e., the process of encoding and transforming information sent via sockets from bytes to some data structure or object and vice-versa). This is due that ZeroMQ and Nanomsg offer a very low-level API as well as a large variety of communication patterns and configurations; therefore, enabling their use for many engineering areas and types of software architectures. Examples of non-robotics middlewares built on top of ZeroMQ are presented in [39,40,41]. An approach enabling interfacing ROS and Unity using ZeroMQ is presented in [42]. Similar to ROS 2.0 with DDS and YARP with the ACE library, NEP abstracts and simplifies the code required to use low-level libraries and robotics frameworks for enabling the easy creation of robotics software architectures.

*NEP is cross-platform*. NEP has been tested in Windows 10 and older Windows versions such as Windows 7 and Windows 8, which are still used by many industries and *end users* as proved in [43]. NEP has also been tested in other OS not fully supported by ROS frameworks such as older versions of OSX as well as older and newest versions of Ubuntu.

*NEP focuses on human-centered projects*. While NEP can be used for academic-oriented applications such as shown in this article, the main focus of NEP is to support *human-centered* design tasks, where non-roboticist are included in the design processes of robots. *Human-centered* approaches are intrinsically multidisciplinary tasks whose main focus is the development of accessible, desirable, intuitive, friendly and usable products able to satisfy human needs and expectations [24,29]. In fact, most researchers in the robotics community mostly focus on *machine-centered* approaches being novelty and performance the main design objectives. As described in [24,25], mature technologies nowadays adopted by the general public have historically switched their design approaches from *machine-centered* to *human-centered*. An example of a human-centered task presented in this article is End-User Development [28].

*NEP is not rosbridge*. NEP does not provide a client implementation using the rosbridge protocol neither is a network protocol for exchanging JSON-encoded ROS topics over *Websockets*. Instead, the main communication protocol used in NEP is the ZeroMQ Message Transport Protocol (ZMTP) (described in [44]).

*Current focus of NEP is not cloud robotics or web teleportation tasks*. Cloud Robotics was initially defined in [45,46] as “an approach to robotics that takes advantage of the Internet as a resource for massive parallel computation and real-time sharing of vast data resources”. An example of a framework for cloud robotics is Rapyuta [47], which is used in the RoboEarth project [48]. Robot Web Tools [15] provides a set of open source modules using the rosbridge protocol as main technology to enable Client/Server messaging of ROS topics over wide area networks WAN. However, cloud robotics is out of the scope of the current objectives of NEP.

## 3. Contributions

The initial version of NEP was presented in [2] as a user-friendly and only Python 2 programming tool for supporting end-user robot programming interfaces. This beta and discontinued version also enabled the re-use of publishers to ROS 1.0. However, the final Application Programming Interface (API) of NEP was changed substantially to enable the use of Python 3 and ROS 2.0. This major update of NEP is the first main contribution of this article. Moreover, a Javascript version of NEP was also developed to enable communication with software enabling the use of web technologies and interfaces using Node.js. This library denoted as *nep.js*, is the second main contribution of this work. The third main contribution of this work is the C# version of NEP which can be used to enable data streaming from novel interactive devices such as *LeapMotion* and *Kinect* as well as modules developed in the game-engine *Unity*. Finally, the fourth main contribution of this article is a latency evaluation and comparative study between proposed NEP libraries (using ZeroMQ) against solutions based only on ROS and the rosbridge suite. These comparisons are performed in both local-host (i.e., two nodes in the same computer) and local area communication (i.e., two nodes in the different computer but in the same wifi network).

### 3.1. NEP for Python

Python is a popular programming language used in the development of Robotics and Artificial Intelligence projects. In many cases, the easy connection of different Python versions is required to enable the creation of intelligent robots. Due to this, NEP has been designed to work in both Python 2 and Python 3, using the same API. NEP for Python also provides a high-level abstraction communication layer which enables the easy switch between similar back-end robotic middlewares and communication libraries. NEP is mostly oriented to support both *Client/Server* and *Publish/Subscribe* communication patterns. Current back-end options supporting the *Client/Server* communication pattern are POSIX sockets and ZeroMQ. Current back-end options supporting the *Publish/Subscribe* communication pattern are ROS 1.0, ROS 2.0, ZeroMQ and Nanomsg. The selection of the desired back-end is defined by the user.

To start using the basic NEP in Python, it is only required to write the command shown in Listing 1 in some console/terminal.

**Listing 1:**
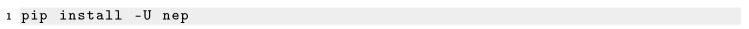
Install NEP in Python.

As an example, the minimal Python code required to create a node publishing data and a node reading this data in NEP is shown in Listing 2 and 3 respectively.

**Listing 2:**
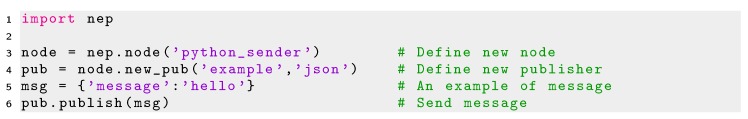
Minimal code using NEP for creating a publisher in Python.

**Listing 3:**
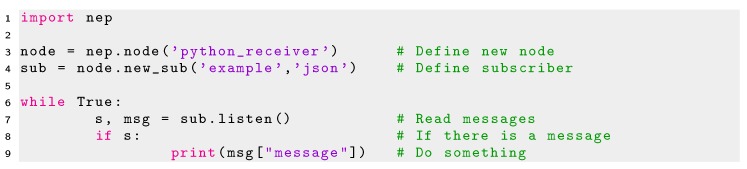
Minimal code using NEP for creating a subscriber in Python.

However, the script of Listing 3 will create a non-ROS re-usable subscriber. In ROS it is required to define special functions denoted as *callbacks* which are only executed when new data arrives at the socket connection. In order to enable the re-use of a NEP subscriber in ROS, the definition of a callback is also required. Minimal code enabling the definition of ROS re-usable callbacks functions in Python is shown in Listing 4.

**Listing 4:**
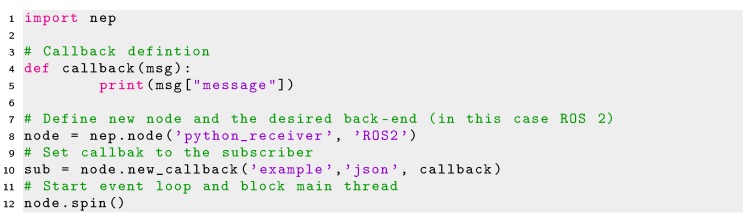
Minimal code using NEP for creating a ROS re-usable subscriber in Python.

NEP was not designed to provide a mechanism that directly connects modules using different protocols and sockets types (e.g., a ZeroMQ subscriber can not read information from a publisher sending data using ROS). This feature is not even available in rosbridge, as messages sent and read from ROS modules to non-ROS compatible modules must be transformed to *Websockets* by the bridge server. Therefore, both the sender and receiver nodes must share the same middleware and protocol. However, users of Python can create their own glue modules enabling the communication between different platforms and robot frameworks using the different back-end options of NEP. In Python NEP offers a high-level API that enables the re-use of code between different communication libraries and robot frameworks as well as simplifies their use and configuration for robot applications. Therefore, the same lines of code used to create a publisher or subscriber in NEP in Python 2 (which is supported by ROS 1.0 and ZeroMQ) can be reused in Python 3 (supported by ZeroMQ and ROS 2.0) just by changing the back-end option to use when creating a NEP node. In the example of Listing 4 the back-end option defined is set in line 8 as *’ROS2’* in the second parameter in the constructor of a new *nep.node* object. This parameter can be defined as *’ROS’*, to use this code in Python 2 and ROS 1.0 or *’ZMQ’* to use this code in both Python 3 or Python 2 using ZeroMQ sockets. When this parameter is not defined, such as Listings 2 and 3, ZeroMQ is used by default.

### 3.2. NEP for Javascript

The integration of JavaScript with robotic systems is relevant to enable the use of IoT technologies, such as cloud-based and web services as well as in the creation of usable Graphical User Interfaces (GUIs) providing better user experiences. NEP bindings for JavaScript and *Node.js*, denoted as *nep.js*, can be used to develop web-based applications able to be executed natively in desktop operating systems and visualized in web-browsers. NEP for JavaScript and *Node.js* can be easily installed writing the command shown in Listing 5 in some console/terminal.

**Listing 5:**
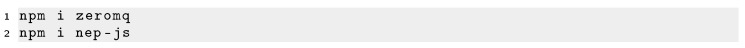
Install NEP in Node.js.

The minimal code required to create a publisher node and subscriber node in Javascript using Node.js are shown in Listings 6 and 7, respectively.

**Listing 6:**
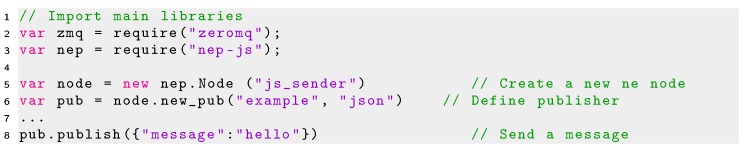
Minimal code using NEP for creating publisher in JavaScript.

**Listing 7:**
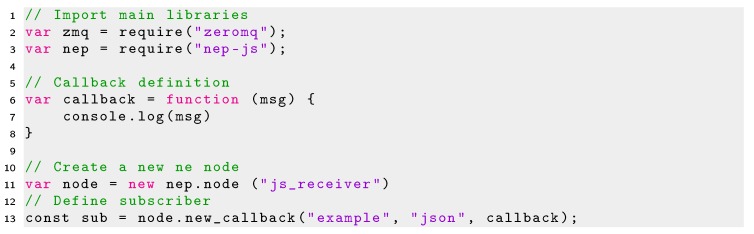
Minimal code using NEP for creating a subscriber in JavaScript.

### 3.3. NEP for C#

C# is a developer-friendly programming language developed by Microsoft. Nowadays C# is one of the most popular programming languages, especially for game developing. Therefore, C# is often the main programming tool in many game engines, virtual and augmented reality systems and interactive sensors and devices (e.g., Cameras, Virtual Reality headsets, Kinect and Leap motion). The integration of many of these tools with robotic systems is often relevant for many researchers in robotics and biomechanics. A few examples can be seen in [49,50,51,52,53,54]. In some cases, these tools are only officially supported or work better in Windows machines (in particular those products developed by Microsoft). NEP offers bindings for C# projects using Visual Studio. These bindings can be easily installed using *Nuget*, which is the official package manager for Microsoft development platforms. For this, users just require to write the commands of Listing 8 in the *Package Manager Console* of Visual Studio.

**Listing 8:**
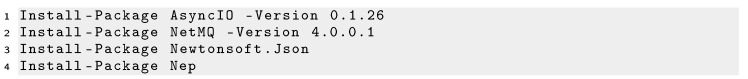
Install NEP in C#.

The minimal code required to create a publisher node and subscriber node in C# is shown in Listings 9 and 10, respectively.

**Listing 9:**
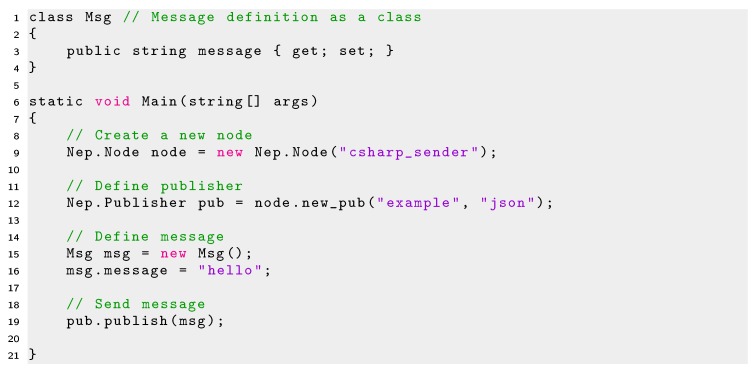
Minimal code using NEP for creating publisher in C#.

Because JSON objects in C# can not be implemented directly, NEP messages must be defined in a class, such as shown in lines 1-4 of Listing 9. In this example, the class *Mgs* is used to define the messages to send. In this class a JSON (*key*,*value*) pair is defined in line 3. The *key* element of the JSON pair will have the name of *message* and the *value* element will be a string. Then, the message to send is created as an object of the *Mgs* class and filled with the string *“hello”* in lines 15 and 16 respectively. Finally, the message is sent in line 19.

**Listing 10:**
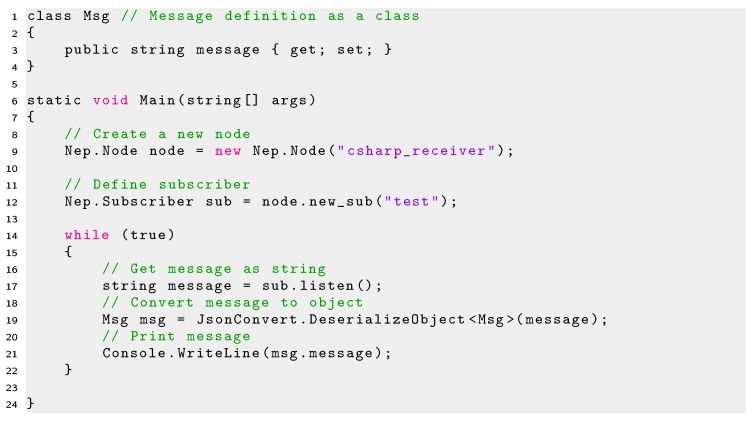
Minimal code using NEP for creating a subscriber in C#.

Similar to the Publisher case, a Subscriber requires the definition of the message to receive as a C# class. Messages in NEP for C# are obtained as strings and must be converted to JSON values using the Json.NET library [55], as shown in line 19 of Listing 10.

## 4. Real-World User Cases

Rather than presenting sample examples tested in laboratory settings of possible applications using NEP, this section briefly describes user cases using NEP for the creation of real-world and complex IoT-aided HRI applications. We briefly present an overview of the software architectures created as well as the main objectives for each use case. However, deeper explanations of experimental protocols, deep learning, control, and cognitive algorithms integrated as well as relevant findings and results of the presented research projects are out of the scope of this article.

### 4.1. Children–Robot Interaction and End-User Development

**Figure 2:**
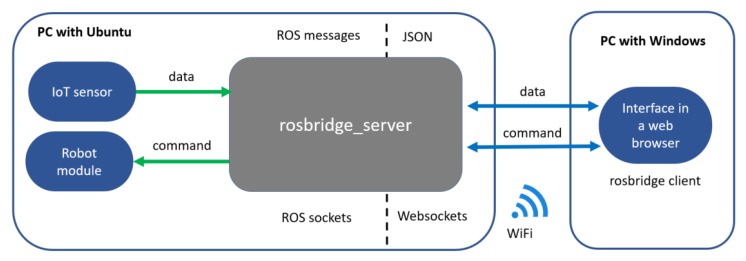
Simple application using a bridge server to connect Robotics and IoT components. The user interface and robot components are executed in different machines. This can introduce delays due to saturation of the WiFi connection and robustness issues of the Client/Server pattern.

**Figure 3:**
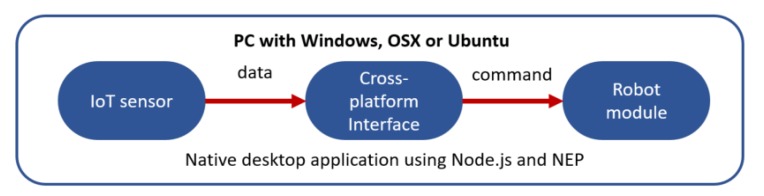
Simple application using Node Primitives (NEP) to connect robot and IoT components in a cross-platform approach. All modules can be executed in the same machine using the Publish/Subscribe pattern; therefore, solving delay issues and improving robustness.

One of the main focuses of NEP is to help in the development of cross-platform End-User Development (EUD), control, and monitoring interfaces [11]. NEP is the main communication tool of the Robot Interface from Zero Experience (RIZE), which is a novel EUD framework for robotics. Unlike similar solutions, RIZE has been designed to be cross-platform, distributed, modular robot-independent and easy-to-install. Initial prototypes of RIZE have been used for developing several demos and real-world applications supporting *end users*. Relevant examples are shown in [1,2]. RIZE is a desktop application developed with modern web technologies, such as *Javascript*, *node.js*, *HTML*, *CSS*, and *Vue.js*. To enable the communication between RIZE and external IoT and robotics modules, the *nep.js* (described in Section 3.2) library is used. Figure 2 and Figure 3 shows a minimal representation using a software architecture enabling EUD and control of robots using a bridge server and NEP, respectively. In order to simplify the explanation these architectures are composed by threes main modules: (i) a sensory module that streams robot perceptual data, (ii) a user interfaces using web technologies that enable the visualization and processing of data and (iii) a robot control module that is in charge to receive and execute the control commands sent by the user interface. Unlike approaches for EUD using rosbridge, observed in Figure 2 and implemented in [56], our approach also enables the execution of robot and IoT modules from the same computer (Figure 3). These modules can be connected using ROS 1.0 modules (using Ubuntu 14 and 16), ROS 2.0 (using Ubuntu 16 and 18, Windows 10 and OSX 10.13 and 10.14) or ZeroMQ (in almost all popular and modern desktop and mobile operating systems). Moreover, the use of ZeroMQ in NEP enables that IoT and robot modules written in almost all programming languages can be directly integrated into the software architecture using high-performance socket communication and the *Publish/Subscribe* pattern.

**Figure 4:**
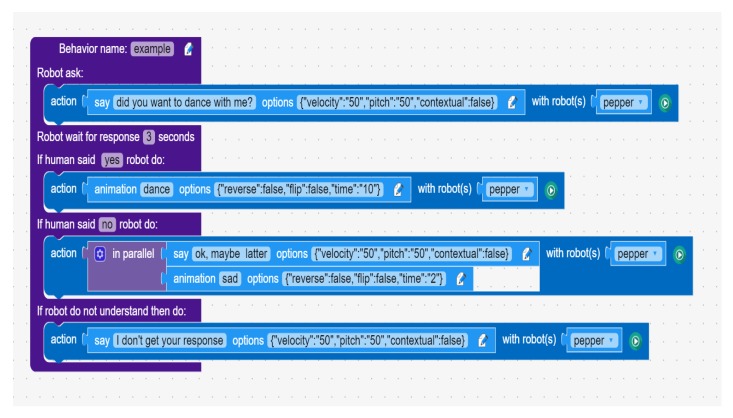
Example of a programming blocks in the Robot Interface from Zero Experience (RIZE) enabling interaction with humans.

**Figure 5:**
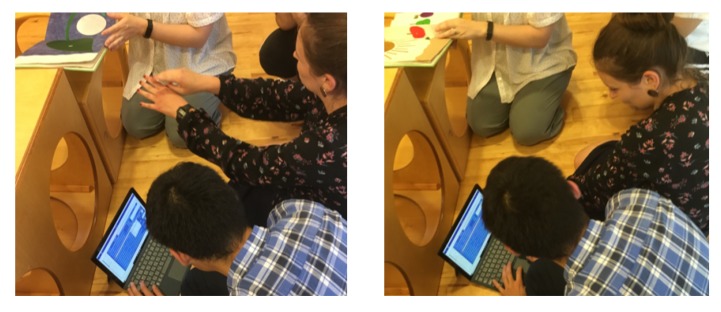
Example of real end users designing and programming a “*in the wild*” Human–Robot Interaction (HRI) scenario using current prototype of the RIZE robot End-User Development (EUD) interface for social robots.

**Figure 6:**
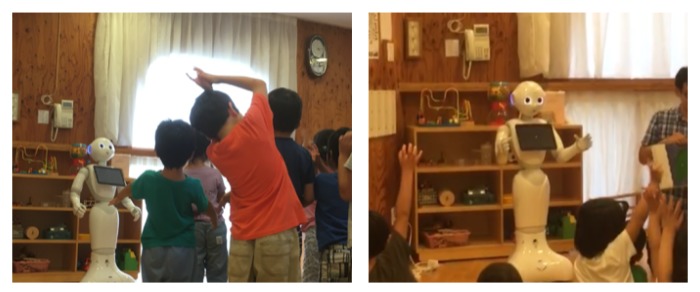
Example of a Children Robot Interaction ”*in the wild*” application developed by end users using RIZE and Pepper robot.

The main programming interface developed in RIZE uses a combination of forms (i.e., buttons, sliders, icons, textareas, and other visual components) and a block-based Visual Programming Language (VPL) integrating the Google Blockly library [44] for enabling the creation of real-world applications using social robots. An example of some programming elements provided in RIZE is shown in Figure 4. In these types of programming environments, users can drag and drop a set of visual elements that represent robot behavioral blocks. More advanced features, such as the design and the Human-Computer Interactions aspects used in the development of this interface (e.g., usability guidelines) are out of the scope of this work and will be reported in a posterior research article in its final and public version. However, some prototypes showing previous designs of the RIZE robot programming environment are shown in [1,2]. Details of the algorithms and the design of relevant visual programming components used to enable *end users* to develop robot applications are described in [1,2,11]. RIZE mostly focuses on enabling the easy and intuitive development and integration of real-world robot applications by adult *end users*. Figure 5 shows real *end users* using RIZE to design and execute a Children–Robot Interaction application [1]. Images showing the execution of the application developed by these *end users* are shown in Figure 6. A recent definition of EUD considers this approach as a sociotechnical activity where users can develop all software and hardware systems that they use in their everyday life [28,57]; therefore, enabling the independence of the owners of the problems (i.e., *end users*) from the high-tech scribes (i.e., programmers and engineers) [58]. Therefore, EUD not only focuses on the program creation phases, such as End-User Programming (EUP), but also in those methods and tools able to support the entire software development lifecycle. This requires reaching independence from high-tech scribes (i.e., experts in programming or engineers) during the use, re-design, configuration, and extension of the software artifacts at use time [28]. One of the main motivations to build NEP is to create a communication framework that does not require programming experience for their use and installation and in this way supporting EUD. For these tasks ROS frameworks present many barriers to the *end users*. In [59] creators of TiViPE [60], an state-of-art EUD tool for social robotics, have reported to critical issues of ROS for EUD: (i) most of the *end users* are Windows users and require easy-to-install tool; and (ii) *end users* will hardly understand (without training) many of the concepts required to use ROS. These barriers are due to their *machine-centered* and academic-oriented focus. In order to use ROS, users require to acquire experience using command-line interfaces (rarely presented in *end users*) as well as deal with complex and many installations of third-party libraries (especially in Windows). With NEP, interfaces as RIZE can be used, installed and configured without any help of high-tech scribes. This is because RIZE can be executed natively in Windows, OSX, and Linux without ROS based tools. This approach differs from interfaces using rosbridge, which are dependent on the rosbridge server and are often configured and launched in laboratory settings by high-tech scribes. An example is Robokol [56], which highlighted the poor communication performance of rosbridge for enabling EUD tasks with social robots. Moreover, approaches used in RIZE can be also applied to other programming paradigms such as the Science, Technology, Engineering, and Mathematics (STEM) for supporting both ROS-enable and non-ROS-enable robots. As shown in the application of Section 4.2 nodes using NEP can be used as a glue library for high-performance communication between ROS robots running in Ubuntu and the end-user computers in Windows.

### 4.2. Human–Robot Interaction on a International Robot Exposition

**Figure 7:**
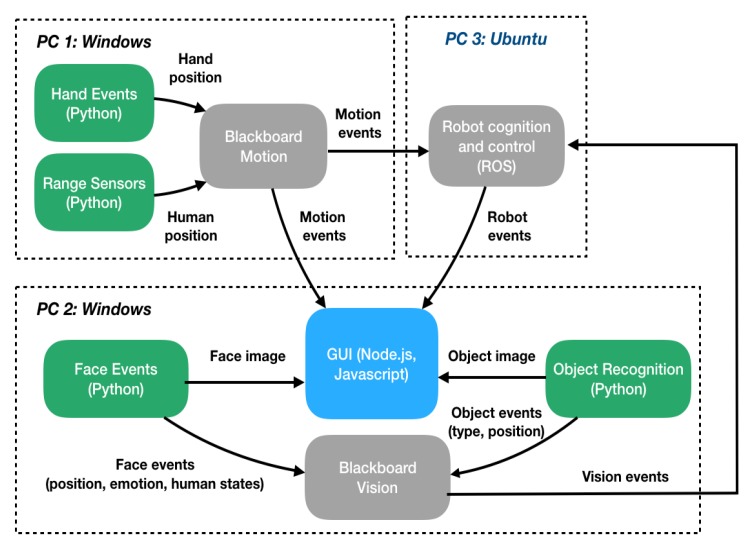
General software architecture of the HRI application performed in the International Robot Exhibition (IREX) 2019.

This section briefly explains the software architecture developed using NEP and ROS for enabling HRI between an industrial robot and the visitors of an international robot exposition. The simplified software architecture of this application is shown in Figure 7. This application required the integration of several sensory devices, computational expensive Deep Learning algorithms, as well as cognitive and robot control modules. Due to the complexity of the system, this architecture was distributed in 3 different computers. Modules in this architecture are mainly connected using the NEP libraries presented in this article.

The PC 2 is composed of 4 modules. The module denoted as *Face States* is composed of a set of Python 3 nodes that uses the images obtained from the camera in the head of the robot to obtain the position, emotions and states (i.e., is human interested or distracted) of the closest human to the robot. The *Object Recognition* module uses the cameras in the robot’s hands to recognize and localize objects organized in a layout and for manipulation purposes. Information from these two perceptual modules is sent to another *Blackboard* node denoted as *Blackboard Vision*. Images after being processed using OpenCV are sent using a NEP publisher to a GUI designed to display relevant information to users. This GUI was developed using modern web technologies and Javascript libraries, such as Node.js, HTML, CSS, and Vue.js. This module uses output images from the *Face States* module to display the current emotions and states of the human interacting with the robot. Output images obtained from the *Object Recognition* module are used to show which is the current manipulation objective of the robot. The programmed interactive scenario includes some playful activities requiring the Leap Motion information. These two interactive activities are: (i) *hand tracking*, where the robot hand position is remotely controlled by humans; and (ii) *selection of an object*, where a human selects the desired object (a chocolate, a pencil or an eraser) in a shop window by finger pointing. This object is later given by the robot to the human as a gift if a human is detected as happy and interested. Leap Motion information is streamed by the *Blackboard Motion* in PC 1 and read by the other two computers in the network for helping in the decision-making process (in PC 3) and visualize objects selected by humans in the GUI of PC 2. Finally, the PC 3 is a Ubuntu PC with ROS 1.0 installed. The main objective of the nodes in this PC is to perform high-level decision making and low-level robot tasks. On the one hand, decision making is performed using the data obtained from the *Blackboard* systems in PC 1 and PC 2. On the other hand, robot movements are performed using ROS libraries for direct and inverse kinematics as well as path planning. Module in PC 3 also sends the interaction and robot status as messages to the GUI in PC 2 to update the elements displayed in the interface.

**Figure 8:**
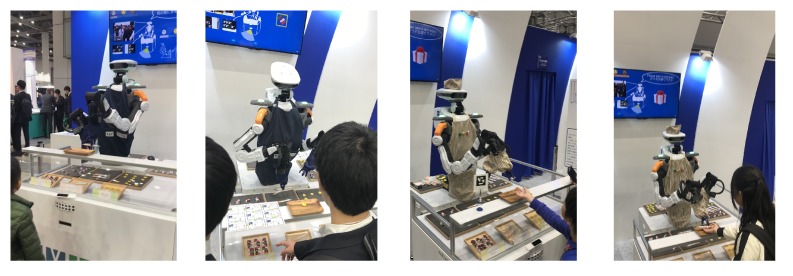
Example of interaction between a ROS-based robot and humans in a international robot exposition.

Figure 8 shows images of the Open NextStage robot performing pick-and-place and HRI tasks in a real-world setting. This robot using the proposed software architecture developed with NEP and ROS was used for a demonstration in the IREX International Robot Exhibition 2019, which was held in Tokyo, Japan [61].

## 5. Performance Evaluation

In Section 4, we prove the technological suitability and applicability of NEP for supporting *human-centered* and IoT-aided Human–Robot Interaction research projects where end users of robots are included in the design of their own applications. The study presented in this section focused on the discoverer suitability of NEP for supporting *machine-centered* projects connecting nodes in both local-host and a LAN network.

### 5.1. Research Questions and Experimental Protocol

The research question (RQ) guiding this study is defined as follows: *Is the performance of NEP suitable for academic-oriented projects requiring low latency communication between nodes written in different programming languages and/or different computers?*

In order to answer the proposed research question, a latency and comparative study against a state-of-art solution (the official rosbridge suite) was performed. This study focuses on two main scenarios: Remote communication in LAN and local-host communication of nodes. One the one hand, scenario (R-remote) is used to discover the suitability of NEP for messaging between 2 nodes launched in different computers connected to the same Wifi router. On the other hand, scenario (L-local) is used to discover the suitability of NEP for messaging between 2 nodes executed in the same computer but written in different programming languages. In this scenario, one of these nodes should be written in a programming language without the ROS official client library. For both scenarios, A-remote and B-local, we use string type messages of different sizes. The range of the transferred data size is 16 KB to 1 MB for R-remote scenario and 256K to 8M for the L-local scenario. A dedicated Wifi router where only the computers used in this study are connected must be used. Moreover, no additional processes must be executed in both computers as well as not an Internet connection must be done when executing the experiments. This is done in order to minimize latency due to external processes in these computers. For this study round-trip latency (i.e., time from the sender to the receiver in other programming language plus the time from the destination back to the sender) is measured.

### 5.2. Experimental Settings

Scenario R-remote is composed of two computers identified as *Machine1* and *Machine2*. The computer used for *Machine1* is a Laptop Dell Inspiron 14 with an Intel Core i7-7500U at 2.7GHz, 8 GB of RAM and Ubuntu 16 installed. The computer used for *Machine1* is a Laptop MSI GF63 8RD with an Intel Core i7-8750H at 2.2 GHz, 16 GB of RAM and Windows 10 installed. NEP 0.5.3.5, as well as ROS Kinetic with the official rosbridge suite, were installed in *Machine 1*. In *Machine 2* NEP 0.5.3.5 and the Python ROS Bridge library in version 0.7.1 were installed. Wi-Fi connection is performed using a low-cost and portable TP-link TL-WR802N router. Nodes in *Machine1*, which are identified as *NEP M1-1* (for a node using NEP) and *ROS M1-1* (for a node using ROS) are written in Python 2. Node in *Machine2*, which are identified as *NEP M2-2* (for a node using NEP) and *Bridge M2-2* (for a node using the Python ROS Bridge library) are written in Python 3. Communication is done in two cases: NEP-RM1 with NEP-RM2 and ROS RM1 with Bridge-RM2.

Scenario L-local was executed in the computer identified as *Machine 1* in the A-remote scenario. Communication for this scenario is performed between nodes written in Python 2 and Node.js (Javascript). Nodes in this scenario are identified as *NEP-L* (for a node using NEP), *ROS-L* (for a node using ROS), *nepjs-L* (for a node using nep.js), *rosnodejs-L* (for a node using rosnodejs). Communication is done in two cases: NEP-L with nepjs-L and ROS-L with rosnodejs-L.

### 5.3. Results and Discussion

**Figure 9:**
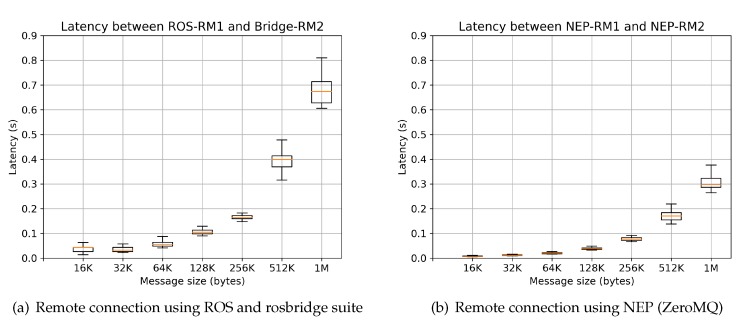
Latency results in scenario R-Remote; comparisons between NEP and ROS-rosbridge using nodes written in Python 2 and Python 3 executed in different machines, which are connected over the same Wifi network.

**Figure 10:**
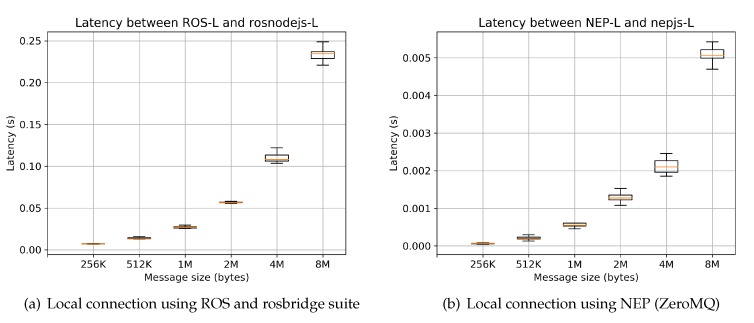
Latency results on scenario L-local; comparisons between NEP and ROS-rosbridge using nodes written in Python 2 and Node.js executed in the same computer.

Results from comparative scenarios R-remote and L-remote are shown in Figure 9 and Figure 10, respectively. These values are obtained after obtaining 200 samples for each message size. Figure 9 shows the case when two nodes executed in different computers are required to be connected. This figure clearly shows that the proposed approach using NEP over ZeroMQ as a back-end option outperforms latency results of ROS using the official rosbridge suite. Moreover, results in local-host of NEP highly outperform latency results reaching by ROS and the rosbridge suite. As mentioned in [62] communication using rosbridge to communicate ROS with other external modules “incurs in significant overhead due to the rosbridge transaction”. With NEP this problem is highly reduced for both local and remote (in LAN) scenarios. These results ask the proposed research question, by proving the suitability of NEP framework for its use in academic-oriented projects.

## 6. Conclusions and Future Work

In this article, we presented the Python, C# and Javascript libraries of the NEP robot programming Framework. The presented software enables the integration of IoT sensors with robotics systems as well as the creation of cross-platform and web-based applications executed in desktop environments. We prove the technological suitability of NEP briefly describing the general software architectures of two successful real-world applications where robots require to interact with humans in “*the wild*” settings rather than laboratories. As shown in one of these applications, NEP can be used as a glue software enabling the integration of state-of-art robotics research frameworks and modules, with *human-centered* software interfaces. NEP have also been designed to be easy-to-implemented by the application programmer and easy-to-install by the *end user*. The proposed approach does not use a bridge server. Instead, NEP directly uses the *Publish/Subscribe* pattern to communicate processes in the same computer or multiple computers in the local IoT network. However, NEP has not been designed to fully replace options using *Websockets* such as rosbridge or *yarp.js*, which can be used to send and receive messages over wide area networks (WAN). However, many research and real-world applications can only require local area networks (LAN), especially when internet access is limited or not robust enough. Moreover, ZeroMQ sockets are present in almost all programming languages and devices such as Android and iOS smartphones. Therefore, supporting a new programming language becomes a trivial issue. Current NEP libraries are accessible to developers as free to use and open source in [63]. Finally, the presented study proved technically superiority (lower latency) of the proposed approach in both local-host and LAN communication against the state-of-art approach for communicating robotics written in programming languages and launched form operating systems not fully supported by ROS. Findings indicate that NEP can be used not only to create usable software artifacts for robotics in *human-centered* research tasks and applications, but also as a low latency option for *academic-oriented* projects connecting ROS with non-ROS enabled modules or devices.
